# Survey of probable synergism between melittin and ciprofloxacin, rifampicin, and chloramphenicol against multidrug-resistant *Pseudomonas aeruginosa*

**DOI:** 10.3389/fmicb.2024.1480299

**Published:** 2024-11-21

**Authors:** Mahsa Sedaghati, Reza Akbari, Lida Lotfollahi Hagghi, Saber Yousefi, Tahere Mesbahi, Mahdieh Delfi

**Affiliations:** Department of Microbiology and Virology, School of Medicine, Urmia University of Medical Sciences, Urmia, Iran

**Keywords:** melittin, antimicrobial peptides, multi drug resistance, anti-biofilm, synergism

## Abstract

**Background:**

The emergence of multidrug-resistant bacteria and also biofilm-associated infections is a great health concern due to the failure of available antibiotics. This has alerted scientists to developing alternative antibiotics. Melittin as an antimicrobial peptide has antibacterial synergistic activity in combining with conventional antibiotics against pathogenic bacteria. Accordingly, this study aimed to assess the synergistic effect of melittin in combination with Ciprofloxacin, Rifampicin, and Chloramphenicol against MDR strains of *P. aeruginosa*.

**Materials and methods:**

Fifty strains of *P. aeruginosa* were isolated from clinical specimens. The antibiotic susceptibility of isolates was evaluated by the disk diffusion method. The MIC and MBC of melittin and melittin-antibiotics combination against isolated strains were examined by microdilution method. The probable synergism between melittin and antibiotics was assayed using the FIC protocol. Time-killing kinetics and anti-biofilm effects of melittin and melittin-antibiotics combination were evaluated using time-kill kinetics and crystal violet staining method, respectively. The toxicity of the melittin-antibiotics combination on the HEK293 cell line was also assessed by the MTT assay method.

**Results:**

Out of 50 isolates of *P. aeruginosa*, 15 strains are considered to be multidrug strains. Among MDR strains of *P. aeruginosa*, 42.85% were resistant to cefepime and ceftazidime and all urine-originate isolates were resistant to cotrimoxazole. A combination of MIC dose of ciprofloxacin and melittin decreased resistance against ciprofloxacin up to 33%. The ciprofloxacin-melittin combination showed a favorable synergism and anti-biofilm effect and was also 30.3% less toxic than melittin alone at 4 μg/ml against the HEK293 cell line. In contrast to ciprofloxacin, with the melittin-rifampicin and melittin-chloramphenicol combinations, an addition effect occurred, respectively, in 86.66 and 53.33% of MDR strains of *P. aeruginosa*.

**Conclusion:**

Combining melittin’s antibacterial and anti-biofilm properties with traditional antibiotics may offer a novel strategy to address antibiotic resistance in *P. aeruginosa*. The simultaneous administration of melittin and ciprofloxacin in a single dose has shown a marked increase in antibacterial effectiveness while minimizing toxicity to the HEK293 cell line. It is advisable to conduct additional research to explore the combined antibacterial effects of melittin and ciprofloxacin in a wider range of clinical samples, animal models, and clinical trial settings.

## 1 Introduction

MDR strains of *P. aeruginosa* are highly resistant Gram-negative pathogens which emerged as a significant pathogen in both developed and developing countries (Čivljak et al., 2014). Such fear is even further once pathogens form biofilms on tissues and medical devices. In this situation, biofilm formation allows bacteria to escape from various clearance mechanisms produced by host and synthetic sources (Batoni et al., 2016; Overhage et al., 2008). Therefore, to overcome, bacterial infections need to be discovered, designed, or developed alternative antimicrobial agents (Annunziato, 2019).

Recently, antimicrobial peptides (AMPs) have been considered by scientists due to their prospective potency, rapid action, and a broad spectrum of activities against gram-negative and -positive bacteria (including multidrug-resistant strains), viruses, fungi, and parasites (Huan et al., 2020). These cationic peptides are produced by most living organisms, including insects, plants, microorganisms, and mammals (Ageitos et al., 2017). Some of the previous studies demonstrated that some AMPs could inhibit or diminish biofilm formation in biofilm-producing bacteria or others show synergism with the convectional antibiotics (Yasir et al., 2018). Notably, some AMPs such as LL-37, lactoferrin, citropin, and melittin are able to suppress biofilm formation and induce the dissolution of existing biofilms (Akbari et al., 2018).

AMPs also can synergize with antibiotics, and in some cases, overcome antibiotic resistance. The use of AMPs to increase the potency of already approved antibiotics appears to be a promising option to combat commonly drug-resistant pathogens (Duong et al., 2021). The synergism between AMPs and conventional antibiotics would be a new rational approach for dealing with biofilm-associated infections. In some studies, the synergism between AMPs and conventional antibiotics has been demonstrated. For example, the human AMP LL-37, and human *β*-defensin 3 (HBD3), have antimicrobial synergy with piperacillin-tazobactam, tigecycline, meropenem, and moxifloxacin. In other examples, antibiotic potency against *Clostridium difficile* is improved when both LL-37 and HBD3 are present (Nuding et al., 2014), and the synergism between LL 17–29 and chloramphenicol is shown against methicillin-resistant *Staphylococcus aureus* and multidrug-resistant *P. aeruginosa* (Rajasekaran et al., 2017). Nisin Z, pediocin, or colistin, and penicillin, ampicillin, or rifampicin, have a synergism effect against antibiotic-resistance *Pseudomonas fluorescens* (Naghmouchi et al., 2012). Also, the synergism between melamine and ciprofloxacin was proven against antibiotic-resistant strains of *P. aeruginosa* (Kampshoff et al., 2019). Synergistic combinations of Polymyxin B (discovered initially as an AMP) with erythromycin and tetracycline have been also shown. In particular, variants of indolizidine synergize with Polymyxin B, tobramycin, gentamycin, and amikacin (Ruden et al., 2019).

Melittin as a highly potent antibacterial peptide having antibacterial, antiviral, anti-inflammatory, and antifibrotic properties (Galdiero et al., 2019), may have a good synergistic effect on killing the bacteria and, also, inhibiting biofilm formation (Ndezo Bisso et al., 2022). The antibacterial activity of Melittin is shown against a wide range of microorganisms, including gram-negative and gram-positive bacteria (Galdiero et al., 2019).

An old antibiotic that is potentially active against some MDR pathogens is chloramphenicol. Chloramphenicol is considered a prototypical broad-spectrum antibiotic and frequently is an antibiotic of choice in the developing world. Since it functions by inhibiting bacterial protein synthesis, chloramphenicol has a very broad spectrum of activity against Gram-positive and Gram-negative bacteria, and anaerobes (Brock, 1961). Chloramphenicol has been abandoned in developed countries due to its association with serious adverse effects including irreversible and fatal aplastic anemia (Murray and Shaw, 1997). However, it is still largely used in the developing world, despite the discovery of new antimicrobials with better activity and fewer side effects, to treat certain types of serious bacterial infections when other antibiotics cannot be used (Terreni et al., 2021).

Rifampicin is a broad-spectrum antibiotic that penetrates well into tissues and abscesses. The development of resistance to rifampicin in bacteria is typically due to a single, but variable, point mutation in its target, the b subunit of bacterial RNA polymerase. Regarding previous studies, Rifampicin could synergize the impact of common antibiotics or antimicrobial peptides against gram-negative bacteria. This antibiotic in combination with colistin, imipenem, meropenem, and doripenem, was effective against MDR *P. aeruginosa* strains with a reduction of the MIC for a better therapeutic level (Hu et al., 2016; Tascini et al., 2004). Also, in the case of the combination of rifampicin with AMPs such as Magainin II and Cecropin A, the same results for standard and clinical strains of *P. aeruginosa* were obtained (Cirioni et al., 2008).

Ciprofloxacin is a synthetic fluoroquinolone and is active against most strains of gram-negative and certain gram-positive pathogens. Based on the WHO (World Health Organization) list of essential medicines, ciprofloxacin is used as the first line of treatment for various infectious diseases, e.g., paratyphoid fever, typhoid fever, acute pyelonephritis, and gastroenteritis (Noergaard et al., 2022). However, because of the cartilage toxicity of Fluoroquinolones, these recommendations do not account for selected populations, e.g., pregnant women and also pediatric. Even though, Fluoroquinolones have effective concentrations in the gastrointestinal tract and have an advantage over *β*-lactam antibiotics and also concentrate in a high amount, intracellularly (Long et al., 2012).

Based on our hypothesis, the side effects of Chloramphenicol, rifampicin, and Ciprofloxacin can be diminished without losing their antibacterial activity, or the spectrum of selected populations can be extended. This aim is achievable by using them in a low concentration as a combined form with antimicrobial peptides. This suggestion originated from our hypothesis that Melittin may penetrate into the bacterial membrane by its alpha-helical conformation due to its amphiphilicity and provide the conditions for the entrance of antibiotics to act synergistically with melittin.

This study aimed to assay the antibacterial synergistic effects of Melittin with conventional antibiotics (Ciprofloxacin, rifampicin, and Chloramphenicol) against MDR strains of *P. aeruginosa* and also the anti-biofilm effects of Melittin with Ciprofloxacin against the mentioned organism.

## 2 Materials and methods

### 2.1 Chemical reagents, antibiotics, media, and bacterial samples

The following chemicals, 3-(4,5-dimethyl-2-thiazolyl)-2,5-diphenyl-2H-tetrazolium bromide (MTT) and dimethyl sulfoxide (DMSO) were purchased from Sigma-Aldrich (St. Louis, MO). RPMI-1640, Fetal bovine serum (FBS), and antibiotics powders were purchased from Sigma-Aldrich (St. Louis). All the disks of antibiotics were purchased from MAST Company (Mast Group, Merseyside, UK). Melittin peptide was purchased from Sigma-Aldrich (with >85% purity) (St. Louis, MO). From November 2016 to March 2017, a total of 50 clinical *P. aeruginosa* isolates were collected from burn patients at Shahid Motahari University Hospital in Urmia, Iran.

### 2.2 Antibiotic susceptibility

#### 2.2.1 Disk diffusion test

Antibiotic susceptibility of *P. aeruginosa* isolates was determined based on CLSI recommendation (Institute CaLS, 2023) using the following antibiotic disks: Cefepime (30 μg), Aztreonam (30 μg), Imipenem (10 μg), Gentamicin (10 μg), Ceftazidime (30 μg), Ciprofloxacin (5 μg), Amikacin (30 μg), Piperacillin/Tazobactam, (100/10 μg), Tobramycin (10 μg), and Trimethoprim/Sulfamethoxazole (1.25 μg/23.75 μg). *P. aeruginosa* ATCC 27853 was also used as control strain. According to the definition of multidrug resistance, the isolates were non-susceptible to at least 1 agent in ≥3 different groups of antibiotics considered as multidrug resistance (MDR) strains (Magiorakos et al., 2012). Based on criteria by the European Centre for Disease Prevention, multidrug resistance in *P. aeruginosa* is defined as testing non-susceptible to at least three of eight defined drug classes (aminoglycosides, carbapenems, cephalosporins, fluoroquinolones, *β*-lactam combination agents, monobactams, polymyxins, and fosfomycin) (McAulay et al., 2021).

### 2.3 Antibacterial activity

#### 2.3.1 Minimum inhibitory concentration

The minimum inhibitory concentration (MIC) of antibiotics: ciprofloxacin, rifampicin, chloramphenicol, and melittin and a combination of melittin–antibiotics: (melittin–ciprofloxacin), (melittin–rifampicin) and (melittin–chloramphenicol) for MDR strains of *P. aeruginosa* were examined using broth microdilution method according to CLSI recommendation (Institute CaLS, 2023).

Briefly, 100 μL of sterile Muller Hinton broth (MHB) is added to each well of the microplate. Then, serial dilutions of the antibiotics, melittin, and a combination of antibiotics–melittin (both at MIC dose) were prepared at various concentrations. The 0.5 McFarland standard suspension (equal to 1.5 × 10^8^ CFU^1^/ml) was adjusted at a wavelength of 625 nm. Then, 100 μL of the bacterial suspension at a final concentration (5 × 10^5^ CFU at the volume of 100 μL) was added to each well and incubated for 16–20 h at a temperature of 37°C. The inhibition of bacterial growth was determined by measuring the absorbance at a wavelength of 625 nm using a microplate spectrophotometer (Epoch-BioTek Co., Winooski, VT) and visual inspection. Sterile MHB and bacterial suspension in MHB without antibiotics were used as negative and positive controls, respectively. *P. aeruginosa* ATCC 27853 served as the control MIC experiment and was repeated twice on all isolates (Kowalska-Krochmal and Dudek-Wicher, 2021).

#### 2.3.2 Minimal bactericidal concentration

Minimum bactericidal concentration (MBC) assay for the antibiotics (ciprofloxacin, rifampicin, and chloramphenicol), and melittin–antibiotics at MIC dose (melittin–ciprofloxacin, melittin–rifampicin, and melittin– chloramphenicol) for MDR strains of *P. aeruginosa* were measured according to the CLSI recommendation (Institute CaLS, 2023).

To perform this test, 10 μL of each well was plated on Mueller Hinton agar and incubated at a temperature of 37°C for 24 h, and the resultant colonies were counted after 18–24 h. The MBCs were determined as the lowest concentration of the peptides that killed 100% of the bacteria.

*P. aeruginosa* ATCC 27853 was used as control. MBC experiments were repeated twice on all isolates.

### 2.4 Time-killing kinetic assays

Time-killing kinetic assays were performed according to the NCCLS guidelines (NCCLS 1999) to measure the time-dependent effect of the combination of melittin with selected antibiotics. According to the MIC method described above, serial dilutions were prepared and the bacterial suspension was added to each well of the microplate. After 1, 3, 6, and 24 h, 10 μL was removed from each well and 1:100 is diluted in sterile Muller Hinton broth and then cultured in Muller Hinton Agar, and incubated at 37°C for 24 h. Colonies were counted and killing kinetics were evaluated (Knaack et al., 2019).

### 2.5 Synergism assay

The synergism and other drug interactions between Melittin and antibiotics (Ciprofloxacin, Rifampicin, and Chloramphenicol) at MIC dose were evaluated against MDR strains of *P. aeruginosa* in a 96-well microplate using MIC base protocol as previously described (Kowalska-Krochmal and Dudek-Wicher, 2021).

In brief, serial dilutions of the combination of antibiotics–melittin (both at MIC dose) were prepared at various concentrations. For this, melittin and antibiotics were mixed at their MIC dose in the first well of the microplate and then serial dilution was prepared in MHB. In the following step, 100 μL of the bacterial suspension at 5 × 10^5^ CFU/ml was added to each well and incubated for 24 h at a temperature of 37°C. Inhibition of bacterial growth was determined by measuring the absorbance at a wavelength of 625 nm using a microplate spectrophotometer (Epoch-BioTek Co.) and visual inspection.

Drug interaction between melittin and antibiotics was calculated using the Fractional inhibitory concentration (FIC) indices as the following formula (Fratini et al., 2017):


$$\begin{array}{l} FIC=\left(\mathrm{MIC}\;\mathrm{drug}\;A\;\mathrm{in combination}/\mathrm{MIC}\;\mathrm{drug}\;A\;\mathrm{alone}\right)\\ \;+\left(\mathrm{MIC}\;\mathrm{drug}\;B\;\mathrm{in combination}/\mathrm{MIC}\;\mathrm{drug}\;B\;\mathrm{alone}\right) \end{array}$$

The FIC results are interpreted as (synergy: *n* ≤ 0.5), (Partial synergy = 0.5 < *n* < 1), (Additive effect: *n* = 1), (Indifferent effect: 1 < *n* < 4), and (Antagonistic effect: 4 ≤ n) (Akbari et al., 2019).

In this protocol, sterile MHB and bacterial suspension in MHB without antibiotics were used as negative and positive controls, respectively. MIC experiments were repeated twice on all isolates. *P. aeruginosa* ATCC 27853 was used as standard strain.

### 2.6 Inhibition of biofilm formation

Three clinical biofilm-forming strains of each *P. aeruginosa* (1 × 10^7^ CFU/200 mL) were cultured in TSB + 0.5% glucose supplemented with a gradient of concentration of melittin, antibiotic (ciprofloxacin) and melittin-antibiotic (at MIC dose) in flat-bottom 96-well microplate and incubated at 37°C for 24 h. The wells were washed three times with PBS solution and air-dried consequently. Absolute methanol (200 μL) was added to wells for biofilm fixation. After 15 min, the solution was aspirated and the plates were allowed to dry at room temperature. The wells were stained with 200 μL of crystal violet (1%) for 5 min and were washed with deionized water three times. Then, they were allowed to dry at 50°C for 30 min. Absolute ethanol (200 μL) was added to each well with shaking at room temperature for 30 min (Shinde et al., 2021). The content of each well was transferred to its equivalent well in another microplate and, finally, the absorbance was measured at 595 nm using a microplate spectrophotometer (Epoch, BioTek Co., Winooski, VT, USA).

For this test, TSB-glucose without bacteria and antibacterial agents was used as the negative control. TSB-glucose-containing bacteria but without peptides or antibiotics was used as positive controls. *P. aeruginosa* (ATCC 19606) was used as a biofilm positive strain.

### 2.7 Cytotoxicity assays

The cytotoxicity of melittin and antibiotics (ciprofloxacin, rifampicin, and chloramphenicol) and melittin-antibiotics combination (melittin-ciprofloxacin, melittin-rifampicin, and melittin-chloramphenicol) (at the MIC dose) was assessed by MTT assay, as previously described (Akbari et al., 2019). The 3 × 10^4^ cells of the HEK293 cell line were cultured in Dulbecco’s Modified Eagle’s medium (DMEM) supplemented with 10% FBS and 1% pen-strep. The cells were then incubated at 37°C, with 5% CO2 and 95% humidity. After a day, the MIC and the synergism dose of the agents (antibiotics, Melittin, and antibiotic-melittin) were set serially in a 96-well microplate and then were added to the cells’ wells and the cell lines incubated at the temperature of 37°C with 5% CO2 and 95% humidity for 24 h. Afterward, 20 μL of MTT solution (5 mg/ml in PBS) along with a fresh culture medium was added to each well and then additionally incubated in a dark place for 4 h. Finally, the supernatant of the wells was drained and 100 μL of DMSO was added to each well and the optical density (OD) of the suspensions was measured at 540 nm (Grela et al., 2018). The percentage of cell death was calculated according to the following formula:


$$\mathrm{Toxicity}\%=\left(1-\frac{\mathrm{mean}\;OD\;\mathrm{of}\;\mathrm{sample}}{\mathrm{mean}\;OD\;\mathrm{of control}}\right)\times 100$$

Viability%=100–toxicity

## 3 Results

### 3.1 Disk diffusion test

According to the disk diffusion experimental data, *P. aeruginosa* strains showed antibiotic resistances of about 42.85, 35.71, 28.75, 25, 25, 28.75, 28.75, and 42.85% against ceftazidime, gentamicin, ciprofloxacin, piperacillin-tazobactam, imipenem, aztreonam, tobramycin, and cefepime, respectively. The highest antibiotic resistance corresponded to ceftazidime and cefepime, and the lowest antibiotic resistance was related to amikacin. Out of 50 clinical *P. aeruginosa* isolates, 15 strains were MDR. Antibiogram patterns of *P. aeruginosa* are summarized in Table 1.

**Table 1:** Antibiogram of *P. aeruginosa.*

Antibiotics	sensitive (%n)	Semi-sensitive (*%*n)	Resistant (%n)
Piperacillin/tazobactam (100/10 μg)	20 (71.42%)	1 (3.5%)	7 (25%)
Ceftazidime (30 μg)	16 (57.14%)	0	12 (42.85%)
Cefepime (30 μg)	16 (57.14%)	0	12 (42.85%)
Aztreonam (30 μg)	20 (71.42%)	0	8 (28.75%)
Imipenem (10 μg)	21 (75%)	0	7 (25%)
Gentamicin (10 μg)	18 (64.28%)	0	10 (35.71%)
Amikacin (30 μg)	24 (85.71%)	0	4 (14.28%)
Tobramycin (10 μg)	20 (71.42%)	0	8 (28.75%)
Ciprofloxacin (5 μg)	20 (71.42%)	0	8 (28.75%)
Cotrimoxazole (25 μg)	0	0	4 (100%)

### 3.2 Antibacterial assay

#### 3.2.1 MIC and MBC

The MIC for melittin-ciprofloxacin in combination form against 15 MDR strains of *P. aeruginosa* were (0.25–4 μg/ml) and (0.125–32 μg/ml), respectively. The MIC values for melittin-rifampicin in combination form in all MDR strains were (1–8 μg/ml) and (0.5–4 μg/ml), respectively. The MIC values for melittin-chloramphenicol were (1–8 μg/ml) and (8–64 μg/ml), respectively. The frequency distributions of MIC and MBC for melittin, ciprofloxacin, rifampicin, and chloramphenicol for MDR strains of *P. aeruginosa* are summarized in Table 2–4.

**Table 2:** Antimicrobial activity of melittin, ciprofloxacin, and melittin-ciprofloxacin.

Strains	Mel (μg/ml)		Cip (μg/ml)		Mel-Cip (μg/ml)		FICi	Interpretation
	MIC	MBC	MIC	MBC	MIC	MBC	Mel-Cip	
6F	4	8	1	2	1–0.25	1–0.25	0.5	Synergy
11F	8	8	1	1	1–0.125	1–0.125	0.25	Synergy
17F	2	4	1	1	0.5–0.25	1–0.5	0.5	Synergy
21F	8	8	1	4	1–0.125	1–0.125	0.25	Synergy
29F	16	>16	16	64	4–4	4–4	0.5	Synergy
33F	2	4	1	1	0.5–0.25	0.5–0.25	0.5	Synergy
35F	2	4	1	1	0.5–0.25	2–1	0.5	Synergy
40F	4	8	1	1	1–0.25	2–0.5	0.5	Synergy
P1	8	16	1	1	1–0.125	2–0.5	0.25	Synergy
P7	16	>16	16	64	4–4	4–4	0.5	Synergy
P9	4	8	8	16	1–2	1–2	0.5	Synergy
P12	2	4	128	>128	0.5–32	1–64	0.5	Synergy
P14	2	2	16	16	0.25–2	0.5–8	0.25	Synergy
P17	4	8	1	1	0.5–0.125	1–0.5	0.25	Synergy
P29SH	8	16	8	8	1–1	2–2	0.25	Synergy
ATCC	16	>16	0.5	1	2–0.062	2–0.062	0.24	Synergy

Mel: melittin; Cip, ciprofloxacin; FICi, fractional inhibitory concentration indices; MIC and MBC for melittin and chloramphenicol in combined form.

**Table 3:** Antimicrobial activity of melittin, rifampin, and melittin-rifampin.

Strains	Mel (μg/ml)		Rif (μg/ml)		Mel-Rif (μg/ml)		FICi	Interpretation
	MIC	MBC	MIC	MBC	MIC	MBC	Mel-Rif	
6F	4	8	2	4	2–1	2–1	1	Addition
11F	8	8	2	4	4–1	4–1	1	Addition
17F	2	4	2	4	1–1	2–2	1	Addition
21F	8	8	2	8	4–1	4–1	1	Addition
29F	16	>16	2	8	4–0.5	2–1	0.5	Synergy
33F	2	4	2	8	1–1	2–2	1	Addition
35F	2	4	2	8	1–1	2–2	1	Addition
40F	4	8	2	16	2–1	4–2	1	Addition
P1	8	16	4	16	4–2	4–2	1	Addition
P7	16	>16	4	4	4–1	8–2	0.5	Synergy
P9	4	8	8	32	2–4	4–8	1	Addition
P12	2	4	4	8	1–2	2–4	1	Addition
P14	2	2	4	16	1–2	1–2	1	Addition
P17	4	8	4	16	2–2	4–4	1	Addition
P29SH	8	16	4	4	4–2	8–4	1	Addition
ATCC	16	>16	4	16	8–2	8–2	1	Addition

Mel, melittin; Rif, rifampicin; FICi, fractional inhibitory concentration indices; MIC and MBC for melittin and Rifampicin in combined form.

**Table 4:** Antimicrobial activity of melittin, chloramphenicol and melittin-chloramphenicol.

Strains	Mel (μg/ml)		Chl (μg/ml)		Mel-Chl (μg/ml)		FICi	Interpretation
	MIC	MBC	MIC	MBC	MIC	MBC	Mel-Chl	
6F	4	8	128	>128	2–64	4–128	1	Addition
11F	8	8	64	>128	8–64	8–128	2	Indifference
17F	2	4	64	>128	2–64	4–128	2	Indifference
21F	8	8	64	>128	8–64	8–64	2	Indifference
29F	16	>16	128	>128	8–64	8–64	1	Addition
33F	2	4	16	64	2–16	2–16	2	Indifference
35F	2	4	16	64	2–16	2–16	2	Indifference
40F	4	8	8	32	4–8	8–8	2	Indifference
P1	8	16	16	64	8–16	8–16	2	Indifference
P7	16	>16	128	>128	8–64	8–64	1	Addition
P9	4	8	64	>128	2–32	2–32	1	Addition
P12	2	4	128	128	1–64	1–64	1	Addition
P14	2	2	128	>128	1–64	2–128	1	Addition
P17	4	8	64	>128	2–32	4–64	1	Addition
P29SH	8	16	16	32	4–8	8–16	1	Addition
ATCC	16	>16	32	128	8–16	8–16	1	Addition

Mel, melittin; Chl, chloramphenicol; FICi, fractional inhibitory concentration indices; MIC and MBC for melittin and rifampicin in combined form.

### 3.3 Time-kill kinetics

**Figure 1:**
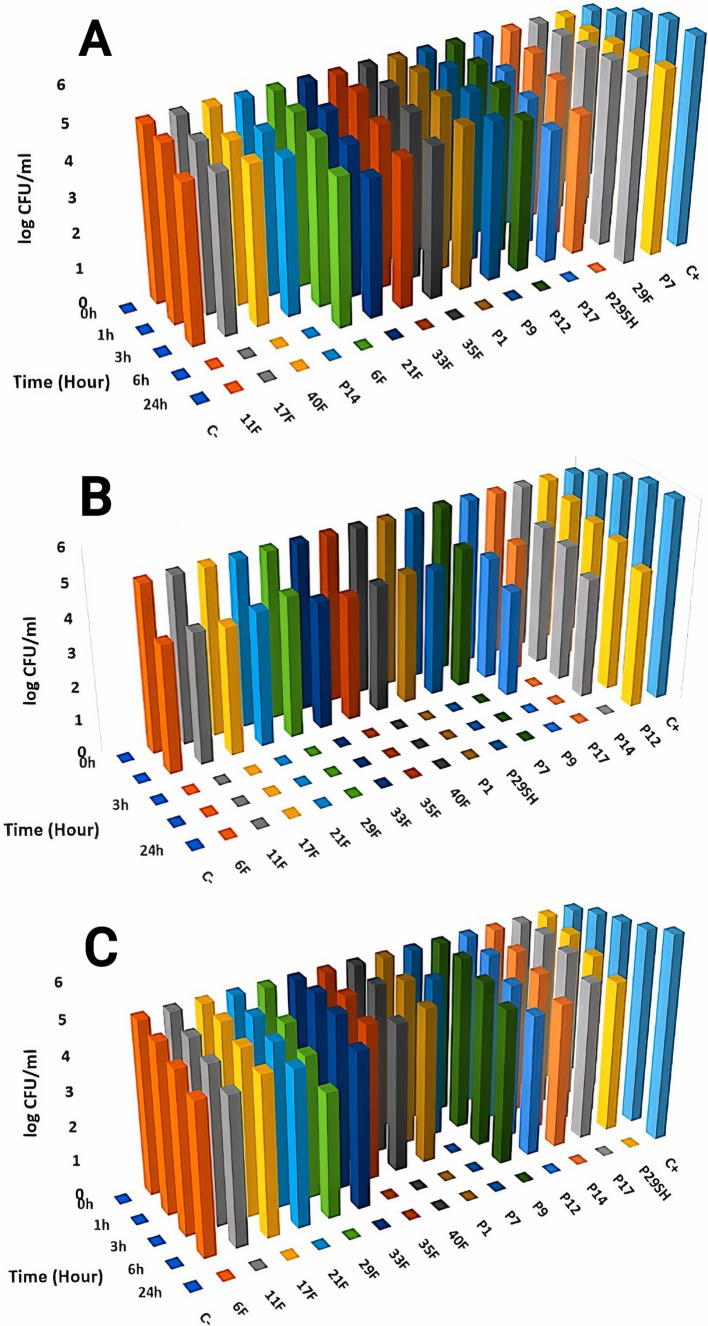
Killing kinetics of melittin (A), ciprofloxacin, (B) and melittin-ciprofloxacin combination **(C)** in MBC dose. C–, negative control; C+, positive control.

**Figure 2:**
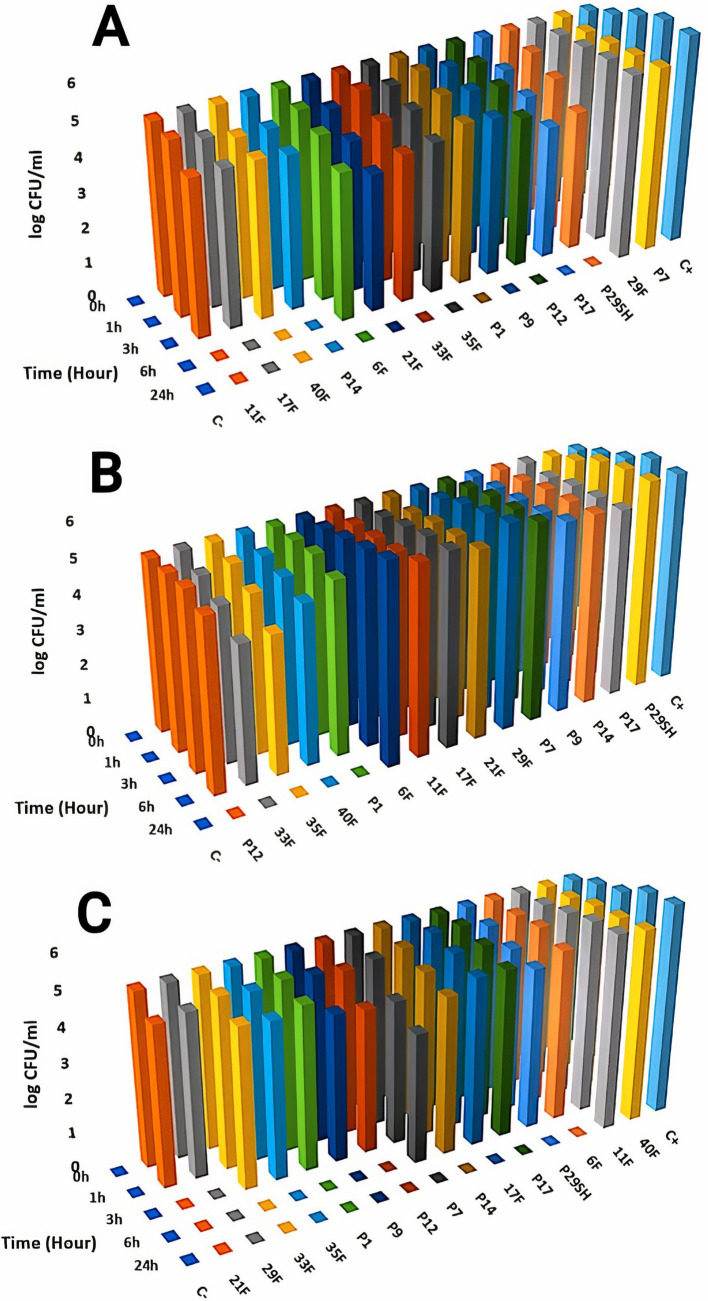
Killing kinetics of melittin (A), chloramphenicol (B), and melittin-chloramphenicol combination (C) in MBC dose. C–, negative control; C+, positive control.

**Figure 3:**
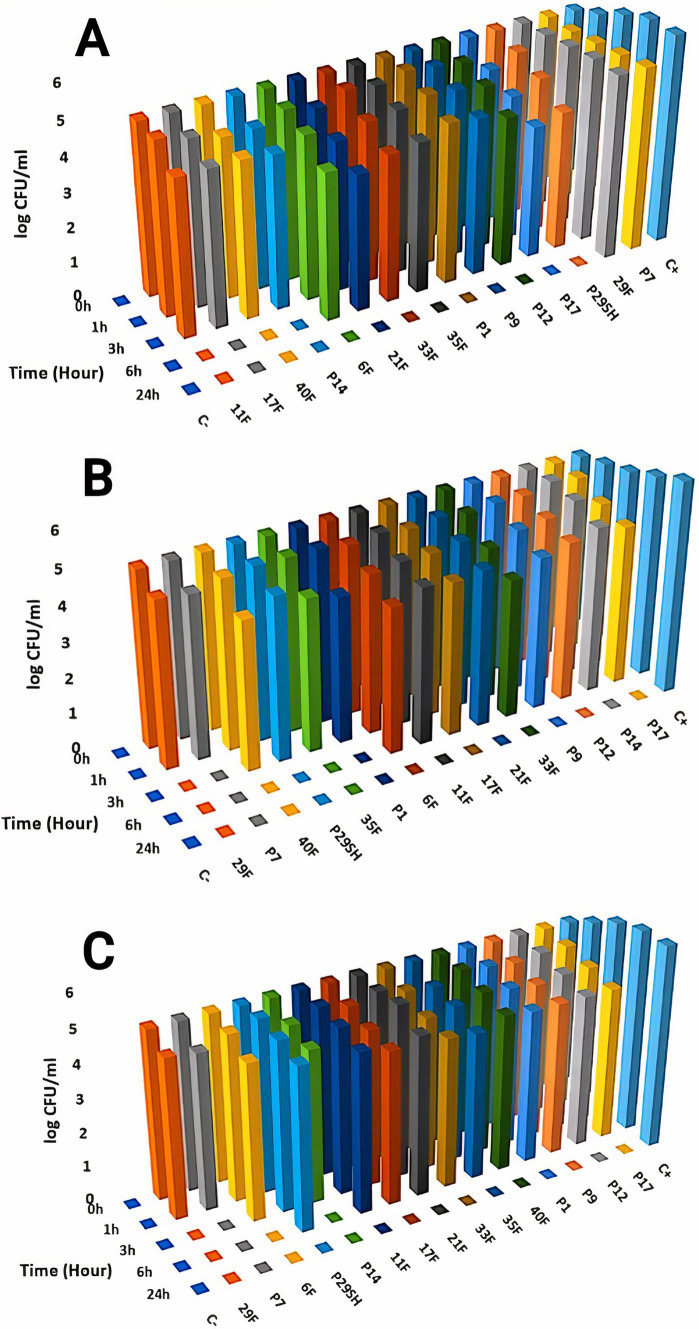
Killing kinetics of melittin (A), rifampin (B), and melittin-rifampin combination (C) in MBC dose. C–, negative control; C+, positive control.

Killing kinetics for melittin and antibiotics (ciprofloxacin, rifampicin and chloramphenicol) were evaluated for MDR *P. aeruginosa* strains (Figures 1–3). Melittin in 8, 2, 4, and 2 μg/ml reduced the logarithm of 11F, 17F, 40F, and P14 strains to zero during 6 h. Ciprofloxacin at a concentration of 1 μg/ml killed all bacterial cells of 6F, 11F, 17F, and 21F strains in 3 h. Rifampicin in 2 and 4 μg/ml reduced the logarithm of 29F and P7 strains to zero in 3 h, respectively. And also, 2 μg/ml at a time of about 6 h, killed 35F and 40F strains. Melittin in melittin-ciprofloxacin form at concentrations of 0.5, 1, and 1 μg/ml reduced, respectively, the logarithm of 35F, 40F, and P1 strains to zero, during 6 h. Also, ciprofloxacin in 0.2, 0.4, and 0.4 μg/ml in a combined form with melittin killed all the same strains of *P. aeruginosa* in the same hour as melittin. Melittin in the combination of melittin-rifampicin at a concentration of 4 μg/ml and rifampicin at 0.5 and 1 μg /ml in 3 h were able to reduce the logarithm of 29F and P7 bacteria to zero. Chloramphenicol in amounts of 16, 16, 8, and 16 μg/ml could reduce the logarithm of 33F, 35F, 40F, and P1 strains to zero in 24 h. Chloramphenicol at a concentration of 64 μg/ml in chloramphenicol-melittin form could reduce the logarithm of 21F and 29F bacteria to zero in 3 h, and melittin in the same combination, in the same hour and at the amount of 8 μg /ml, reduced the logarithm of 21F and 29F strains to zero.

### 3.4 Synergism

FIC indices related to melittin-ciprofloxacin showed a synergism effect in all strains. The addition effect occurred in 86.66 and 53.33% of MDR strains with the melittin-rifampicin and melittin-chloramphenicol combinations, respectively.

### 3.5 Cytotoxicity activity

**Figure 4:**
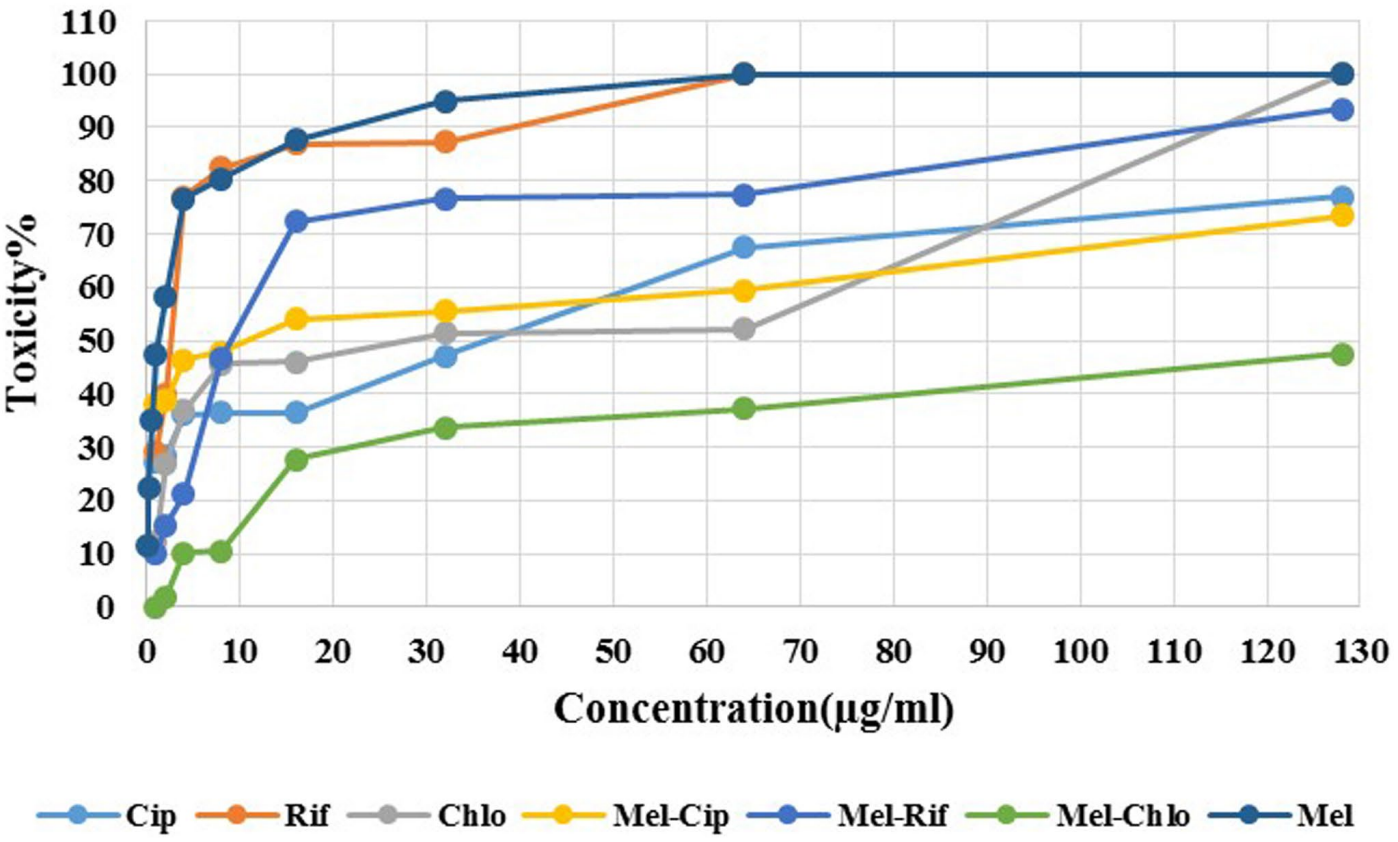
Cytotoxicity of melittin and ciprofloxacin, rifampin and chloramphenicol and melittin-ciprofloxacin, melittin-rifampin, and melittin-chloramphenicol on the HEK293 cell line. Mel, melittin; Cip, ciprofloxacin; Rif, rifampicin; Chl, chloramphenicol.

As Figure 4 depicts, melittin at 1 μg/ml and 0.125 μg/ml (MIC dose) showed approximately 50 and 11.35% toxicity against the HEK293 cell line, respectively. Ciprofloxacin, Rifampicin, and Chloramphenicol at a concentration of 2, 1, and 1 μg/ml, respectively showed 30, 30, and 11% toxicity against the HEK293 cell line. The melittin-ciprofloxacin, melittin-rifampicin, and melittin-chloramphenicol combinations at amounts of 2, 4, and 16 μg/ml had 40, 20%, and more than 30% toxicity against the HEK293 cell line. Melittin at a concentration of 0.062 μg/ml and chloramphenicol at a concentration of 0.5 μg/ml in the melittin-chloramphenicol combination, showed 0% toxicity against the HEK293 cell line. In other concentrations, Melittin-ciprofloxacin at MIC = 0.062–0.5 μg/ml showed 37.95% (AVG) toxicity against the HEK293 cell line. Both groups of melittin-rifampicin and melittin-chloramphenicol at MIC = 0.062–0.5 μg/ml showed 10.21 and 0% toxicity against the HEK293 cell line.

### 3.6 Anti-biofilm assay

**Figure 5:**
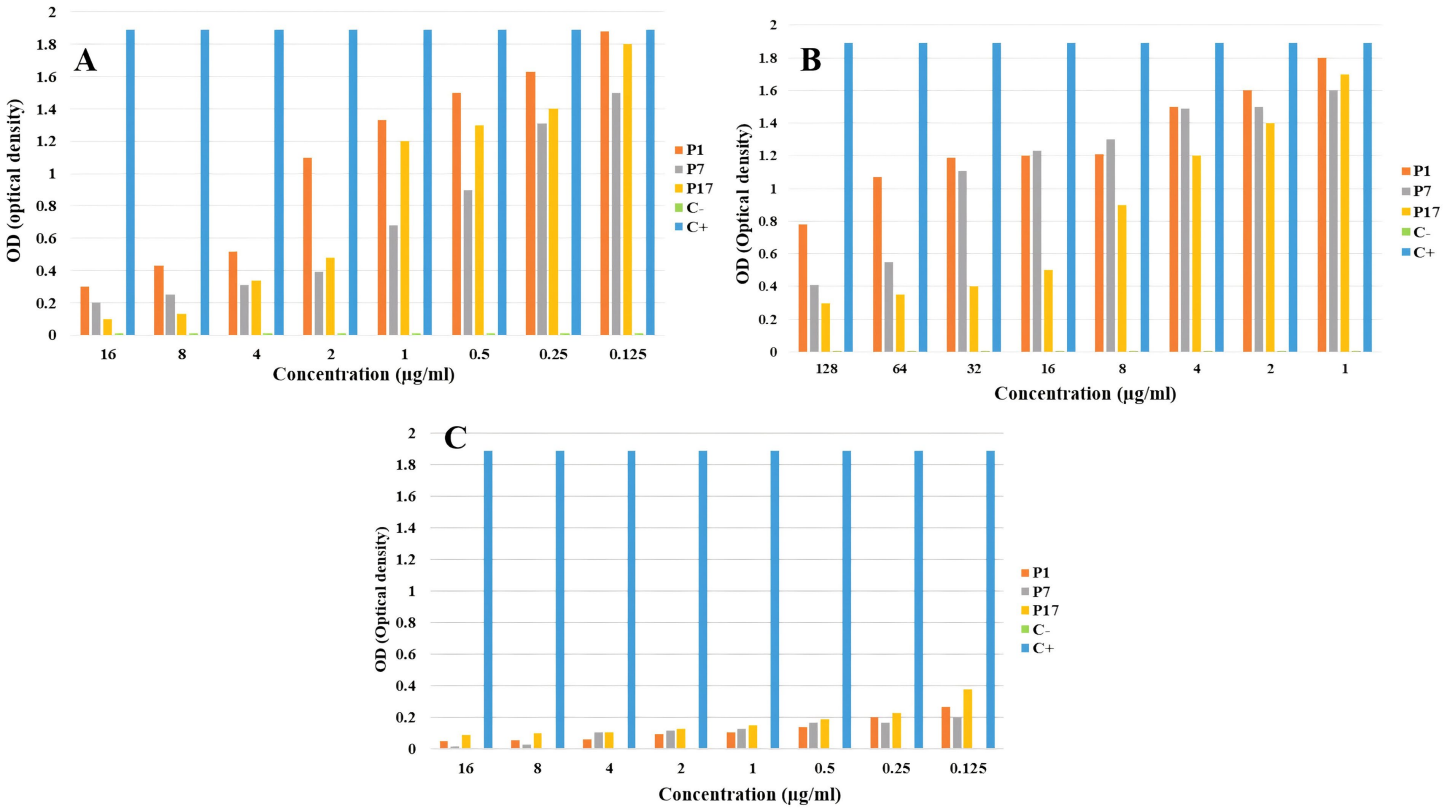
The anti-biofilm effect of melittin (A), ciprofloxacin (B), and melittin-ciprofloxacin combination (C) on *P. aeruginosa* strains.

The results of the anti-biofilm effect showed that the melittin-ciprofloxacin combination at MIC concentration had a better anti-biofilm effect against the biofilm of *P. aeruginosa* than ciprofloxacin at the same concentration. The results are shown in Figure 5.

### 3.7 Therapeutic index (TI)

The therapeutic index of the combination of melittin with rifampicin, chloramphenicol, and ciprofloxacin was calculated based on the ratio of cell toxicity concentration (CTC) against the HEK293 cell line, over the mean minimum inhibitory concentration (GM-MIC).


TI=CTC/GMofMIC

**Figure 6:**
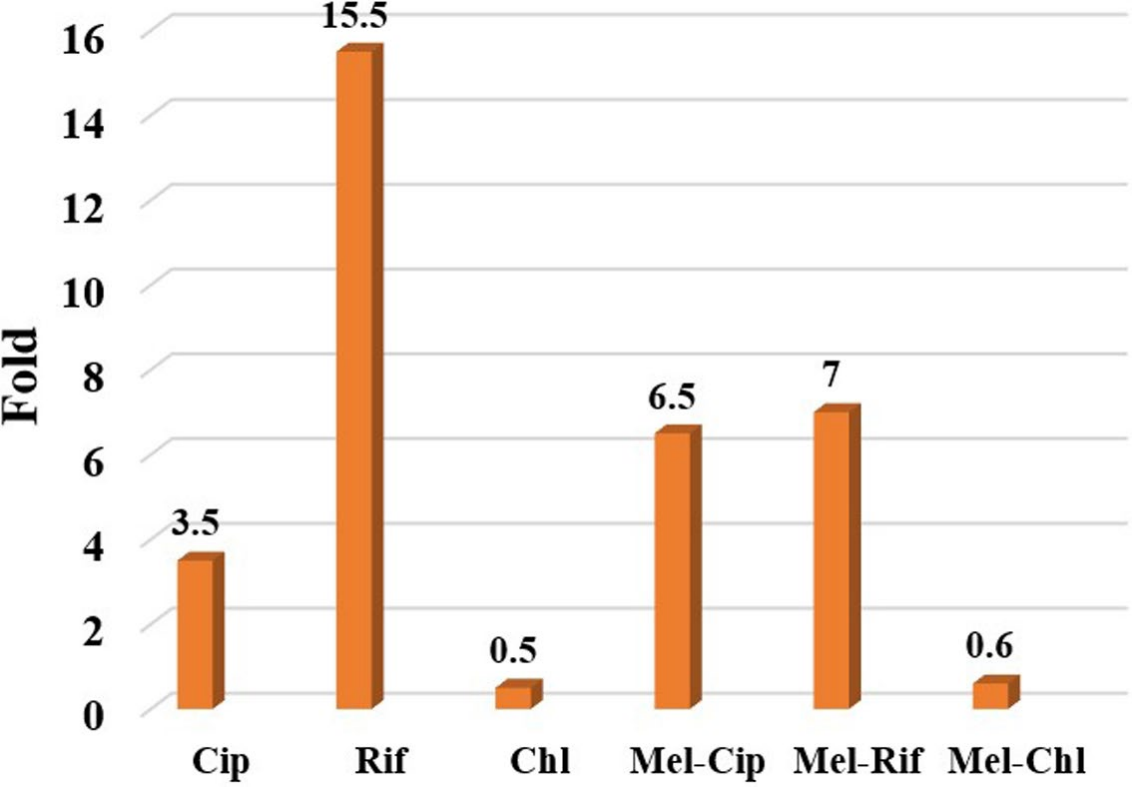
The ratio of improving the therapeutic index of melittin-ciprofloxacin, melittin-rifampin, and melittin-chloramphenicol to melittin.

The results showed that the therapeutic index of melittin-ciprofloxacin, melittin-rifampicin, and melittin-chloramphenicol, were improved, respectively, *ca.* 6.5, and 7 and 0.6-fold in comparison with the melittin alone (Figure 6 and Table 5).

**Table 5:** Therapeutic index of melittin and antibiotics and melittin-antibiotic combination.

	MCTC (μg/ml)	GM MIC (μg/ml) (*n* = 15)	TI (*n* = 15)
Melittin	0.125	6	0.020
Mel-Cip	0.062 + 0.5	1.18 + 3.11	0.13
Mel-Rif	0.062 + 0.5	2.46 + 1.5	0.14
Mel-Chl	0.062 + 0.5	4.13 + 42.66	0.012
Cip	1	13.4	0.07
Rif	1	3.2	0.31
Chl	1	68.8	0.01

GM, Geometric Mean; MCTC, Minimal Cell Toxicity Concentration; TI, Therapeutic index.

## 4 Discussion

Infections with MDR bacteria are a major problem worldwide, and the inability of traditional antibiotics to effectively treat these illnesses has emerged as a significant challenge (Issam et al., 2015). It is necessary to use alternative antibacterial agents. A novel strategy for combating infectious diseases caused by MDR strains of pathogens involves the combination of antimicrobial peptides and conventional antibiotics. Previous studies have successfully demonstrated the existence of a synergistic effect between melittin and some of antibiotics (Akbari et al., 2019; Mirzaei et al., 2023).

According to the findings of this study, the disk diffusion method revealed that 42.85% of *Pseudomonas aeruginosa* strains showed resistance to cefepime and Ceftazidime, and all of the strains isolated from urine samples were resistant to cotrimoxazole. This resistance rate in *P. aeruginosa* strains isolated from urine samples against cotrimoxazole was reported approximately 5.93% based on research conducted by Akif Bayyiğit and his colleague in 2023 in Turkey (Bayyiğit et al., 2023).

In the study by Akbari et al. in 2018, the resistance of *P. aeruginosa* to ceftazidime was reported to be 56% and cefepime was 48% (Akbari et al., 2018), the results of our study were very close to these results. In the study of Safai et al. in 2017 in Hamedan, the resistant *P. aeruginosa* to ceftazidime was reported to be 75% (Safaei et al., 2017). There may be variations in the results of our study compared to the recent study due to differences in the type of clinical isolates utilized and geographical location. In our study the resistance rate of the *P. aeruginosa* strains against ciprofloxacin was 40%. This amount was reported 77.33 and 41.37% in the studies conducted by of Nouri et al. in 2014 in Tabriz, and Moshirian Farahi, et al. in 2018 in Tehran, respectively (Farahi et al., 2018; Nouri et al., 2016). The findings of this study indicate that the Ciprofloxacin-Melittin combination significantly decreases resistance in multidrug-resistant (MDR) *Pseudomonas aeruginosa* strains against ciprofloxacin, with a reduction of 33% in the minimum inhibitory concentration (MIC). In other words, the amount of resistance in all examined strains has decreased by 1.2 times. The synergism effect between melittin and ciprofloxacin was shown in 100% of *P. aeruginosa* strains. This information is consistent with the results of FICI of this study. For example, the melittin-ciprofloxacin combination resulted in a three-fold reduction of MIC values against the multidrug resistant strains 11F, P29SH, and 21F *P. aeruginosa*. Similarly, for the P14 MDR strain, the MIC of melittin-ciprofloxacin was five times lower than that of ciprofloxacin alone. Ciprofloxacin is among the recommended antibiotics by CLSI for treating *P. aeruginosa*-related infections. Numerous studies have assessed its effectiveness against this bacterium (Derakhshan et al., 2020).

The efficacy of rifampicin and chloramphenicol as antimicrobial agents against *P. aeruginosa* has been limitedly studied due to the rapid development of resistance in the bacteria or their potential for significant side effects.

The unfavorable nature of rifampicin for *P. aeruginosa* can be attributed to various factors, including its low permeability through the outer membrane of both *P. aeruginosa* and other Gram-negative bacteria. Additionally, the rapid genetic mutation of bacteria in response to rifampicin usage and the involvement of bacterial efflux pumps further contribute to its ineffectiveness against *P. aeruginosa* (Liu et al., 2020). This study determined the MIC range of rifampicin against drug-resistant strains of *P. aeruginosa* was 2–8 μg/ml (the mean of MIC = 3.2 μg/ml). Numerous studies have demonstrated that the use of rifampicin in combination with other antibiotics or antimicrobial peptides is highly effective against multidrug-resistant (MDR) strains of bacteria, resulting in a significant antibacterial activity.

According to the researches conducted by Giamarellos-Bourboulis et al. (2003), Tascini et al. (2004), Yang et al. (2016), Cirioni et al. (2007), and Cai et al. (2018), it has been found that the use of rifampicin in combination with colistin/polymyxins is effective in controlling multi-drug resistant *P. aeruginosa* strains (Liu et al., 2020). Several studies have confirmed that there is a synergistic effect between rifampicin and certain antimicrobial peptides. In 2020, a study conducted by Jie Liu and colleagues in China explored the potential synergistic effect of Sphistin and Sph12-38 antimicrobial peptides, obtained from mud crabs, in combination with rifampicin against *P. aeruginosa* bacteria (Liu et al., 2020). In our study, rifampicin did not demonstrate satisfactory synergism against *P. aeruginosa* when combined with melittin, despite the findings of Jie Liu. This contradiction shows that rifampin interacts differently in combination with different antimicrobial peptides. Chloramphenicol has limitations in its clinical application against *P. aeruginosa* due to the rapid development of resistance via the efflux pump mechanism (Nitzan and Rushansky, 1981; Oong and Tadi, 2024). Numerous researchers are working to address these challenges to make chloramphenicol more accessible for clinical use. Combining chloramphenicol with different substances is suggested by many scientists to overcome these problems (Chandrasekaran et al., 2020; Lee et al., 2021; Park et al., 2004). Combining chloramphenicol with melittin is one of the methods investigated in this study.

This study found that the MIC range for chloramphenicol against *P. aeruginosa* was 8–128 (with a mean MIC of 68.8). The mean MIC of melittin-chloramphenicol for this bacterium showed a slight decrease of 1.61%. Melittin-chloramphenicol combined showed an additive effect against most MDR *P. aeruginosa* strains (53.33%) and did not show desired synergism against *P. aeruginosa* strains. As before mentioned melittin-rifampicin also showed additive against *P. aeruginosa* strains like melittin-chloramphenicol. The synergistic effect of the melittin-rifampicin combination was detected only in two MDR *P. aeruginosa* strains. As previously mentioned, it is challenging for chloramphenicol and rifampicin to penetrate the outer membrane of *P. aeruginosa*. However, melittin facilitates penetration of these two drugs through the outer membrane of *P. aeruginosa* and, plays a moderate auxiliary role, which caused a slight increase in the anti-*Pseudomonas* activity of these two antibiotics. Unlike chloramphenicol and rifampicin, the effect of ciprofloxacin in combination with melittin against MDR *P. aeruginosa* strains has been very impressive. According to the MIC results of ciprofloxacin, it seems that the penetration of this antibiotic is easily done through the cell wall of *P. aeruginosa*, however, melittin has played a very effective role in increasing the permeability of this antibiotic because the synergistic effect of the ciprofloxacin-melittin combination in most *P. aeruginosa* strains was shown. In the study of Yoonkyung Park et al. in 2004 in South Korea, the combination of chloramphenicol and A3 peptide was evaluated against *P. aeruginosa* and the result of their study showed the existence of a synergistic effect of the combination (Park et al., 2004).

According to the time-kill kinetics findings, melittin combined with chloramphenicol which had shown slight synergistic effects, surprisingly, was the most efficient at quickly eliminating bacteria. In contrast, the melittin-ciprofloxacin combination, despite showing strong synergism, took longer to kill the bacteria. In 6 h, the combination of melittin and chloramphenicol reduced the bacterial count to zero, with mean minimum bactericidal concentrations (MBC) of 4.69 μg/ml. In contrast, chloramphenicol alone at about double dose killed most strains within 24 h. One of the primary challenges with chloramphenicol alone in treating *P. aeruginosa* is the impenetrability of the bacterial wall to the antibiotic (Čivljak et al., 2014). The bacterial cell wall’s permeability is increased when exposed to the chloramphenicol in a chloramphenicol-melittin combination, leading to quicker inhibition of the bacteria. This has shortened the time it takes for chloramphenicol to kill the bacteria. Melittin potentially is effective in pore formation on the cell wall of bacteria and facilitates chloramphenicol entrance through the cell wall.

The killing time of bacteria was approximately the same for both the melittin-rifampicin combination and rifampicin alone.

Although the Ciprofloxacin-melittin combination demonstrated a beneficial synergistic effect against *P. aeruginosa* strains, it exhibited prolonged killing kinetics. Ciprofloxacin-melittin combination with an average MBC (1.6 μg/ml) decreased bacterial cells to zero within 24 h. However, certain strains like 35F and P1 killed within 6 h. One possible explanation is that the concentration of ciprofloxacin decreased when melittin was added. Because ciprofloxacin’s effectiveness is dependent on concentration, it can inhibit the replication and kill bacteria by targeting the DNA gyrase enzyme at low concentrations. At higher concentrations, it can also affect the topoisomerase IV enzyme (Evans-Roberts et al., 2016). When ciprofloxacin is used at a low concentration in combination with melittin, it slows down the inhibition of the bacterial DNA gyrase enzyme. However, when used alone at a high dose, ciprofloxacin inhibits the bacterial DNA gyrase more quickly, resulting in a shorter killing time for the bacteria. Furthermore, the bacterial efflux pump likely plays a significant role in this scenario. The efflux system helps remove some antibiotics from the cell, resulting in a lower concentration inside the cell. This reduced concentration may not be enough to effectively destroy all DNA Gyrase enzymes quickly, leading to the death of the bacteria in a prolonged time. The study on the cytotoxicity of melittin and antibiotics on the HEK293 cell line revealed that combining melittin with antibiotics resulted in significantly lower toxicity levels compared to using melittin or antibiotics alone, likely due to the reduced dosage required. At a concentration of 4 μg/ml, melittin showed a 30.3% decrease in toxicity against the HEK293 cell line when combined with ciprofloxacin, compared to melittin alone at the same concentration. The combination of melittin and chloramphenicol at concentrations of 0.062 μg/ml and 0.5 μg/ml, respectively, did not exhibit any toxicity toward the HEK293 cell line. The toxicity of the combination is reduced by lowering the dosage of melittin when co-prescribed with ciprofloxacin, rifampicin, and chloramphenicol. In our previous study, it found that the combination of melittin-doripenem at a concentration of 0.006 μg/ml had 0.12% toxicity, and the combinations of melittin-doxycycline and melittin-colistin at a concentration of 37 μg/ml exhibited 12.5% toxicity against the HEK293 cell line. This amount of toxicity was much lower than the toxicity of melittin alone which was treated on the HEK293 cell line (Akbari et al., 2018). To better understand the effectiveness of melittin and antibiotics in therapy, it is important to analyze both their cytotoxicity and antimicrobial activity simultaneously. The therapeutic index (TI) is a more suitable index for this analysis. Improving the therapeutic index of an antimicrobial compound will have an effective antimicrobial activity and also minimize side effects on host cells. The study results showed that the therapeutic index of melittin-ciprofloxacin and melittin-rifampicin combinations is 6.5 and 7 times improved than melittin alone. While melittin alone is too toxic for clinical use, there is potential for its combination with antibiotics like ciprofloxacin, rifampicin, and chloramphenicol to be used in clinical settings. This is particularly promising for local treatment against pathogens like *P. aeruginosa*.

The Ciprofloxacin-Melittin combination had an effective anti-biofilm activity against the biofilm-producing strains of *P. aeruginosa*. Biofilm-related infections caused by *P. aeruginosa* are a significant clinical challenge because traditional antibiotics are ineffective in treating them. According to many reports, antimicrobial peptides (AMPs) can be a promising new agent to overcome this problem. AMPs penetrate bacterial biofilm effectively, eliminating planktonic cells within the biofilm structure (Khozani et al., 2019). This study showed that the anti-biofilm effect of melittin and ciprofloxacin is enhanced when used together compared to when either melittin or ciprofloxacin is used alone. Despite using a lower concentration of melittin and ciprofloxacin, the combination was still able to demonstrate significant anti-biofilm activity. According to the findings of this research, the anti-biofilm effectiveness ranking against *P. aeruginosa* biofilm was determined to be as follows: (Melittin-Ciprofloxacin) > Melittin > Ciprofloxacin. This study showed that some strains of *P. aeruginosa*, which are resistant and form biofilms, were not effectively killed by ciprofloxacin alone. However, they were easily stopped by a combination of melittin and ciprofloxacin. According to a 2018 review by Khozani et al., melittin can slowly infiltrate the layers of *P. aeruginosa* biofilm and eliminate the bacteria within by disrupting the bacterial membrane and releasing intracellular contents (Khozani et al., 2019). In 2016, Dosler and colleagues found that melittin, when combined with amikacin, cefepime, ciprofloxacin, colistin, and doripenem, effectively prevented (up to 72%) biofilm formation in strains of *P. aeruginosa*, *Escherichia coli*, and *Klebsiella pneumoniae* (Dosler et al., 2016). In a study conducted by Bardbori et al. in 2018, it was shown that melittin in combination with colistin and imipenem was able to inhibit the biofilm formation in MDR strains of *A. baumannii* (Bardbari et al., 2018). One possible explanation is that melittin breaks down the biofilm network, allowing ciprofloxacin to penetrate the biofilm matrix and reach the bacterial cells more easily. Anti-biofilm effects of melittin and other antimicrobial peptides have also been mentioned in the studies of other researchers. Galdiero and colleagues demonstrated that antimicrobial peptides (AMPs) inhibit biofilm formation through various mechanisms, including disrupting the matrix, binding to DNA, and altering the expression of genes related to biofilm production. These genes include those involved in Pilli production, quorum sensing, flagellum assembly, as well as targeting the cytoplasmic membrane and intracellular components. Additionally, AMPs can degrade signaling molecules like ppGpp and reduce the expression of genes responsible for intracellular polysaccharide adhesion. They have demonstrated that it is easier to prevent the formation of biofilm in its early stages than to inhibit a mature biofilm (Galdiero et al., 2019). In summary, the findings of this study demonstrated that melittin, in combination with ciprofloxacin, significantly reduces the formation of biofilm and the survival of bacteria within the biofilm in a concentration-dependent manner.

## 5 Conclusion

The spread of MDR strains of *P. aeruginosa* has becomes a global challenge. The infections caused by these strains require immediate development of new antibacterial agents or inhibition strategies. Antimicrobial peptides are promising new compounds that may act as alternative antibacterial agents for the treatment of infectious diseases. A novel approach to combatting multidrug-resistant strains of pathogens involves combining AMPs with traditional antibiotics. Several studies demonstrated Melittin of bee venom as natural AMP with the combination of antibiotics shows a favor synergistic effect. In this study, it was found that the strong antimicrobial synergistic effect of the melittin-ciprofloxacin combination successfully eradicated all multidrug resistant strains of *P. aeruginosa* in a time of less than 24 h. Furthermore, the combination of melittin and ciprofloxacin demonstrated a notable ability to combat biofilm-producing strains of *P. aeruginosa*. It appears to be a promising option for treating biofilm infections caused by drug-resistant strains of *P. aeruginosa*. The melittin-rifampicin combination, with a therapeutic index 7 times higher than melittin alone, is the most highly recommended combination for inhibiting drug-resistant strains of *P. aeruginosa.* The combination of melittin and chloramphenicol effectively eliminates multidrug resistant strains of *P. aeruginosa* at a low concentration and for a short duration (6–24 h). The reduced concentration of chloramphenicol when combined with melittin likely helps to alleviate common side effects of chloramphenicol. Ultimately, the melittin-ciprofloxacin, melittin-chloramphenicol, and melittin-rifampicin combinations show promising antibacterial activity and minimal toxicity, making them a practicable option for treating infections caused by drug-resistant strains of *P. aeruginosa*. Additional research is necessary to assess the potential of these compounds for drug development, including analyzing clinical strains, conducting toxicity assessments, experimental infection studies in animal models, and clinical trials.

## Data Availability

The original contributions presented in the study are included in the article/supplementary material, further inquiries can be directed to the corresponding author.

## Footnotes

^1^Colony Forming Unit.
